# Pre-admission functional status impacts the performance of the APACHE IV model of mortality prediction in critically ill patients

**DOI:** 10.1186/s13054-017-1688-z

**Published:** 2017-05-15

**Authors:** James S. Krinsley, Thomas Wasser, Gina Kang, Sean M. Bagshaw

**Affiliations:** 1 Division of Critical Care, Department of Medicine, Stamford Hospital, Columbia University College of Physicians and Surgeons, 1 Hospital Plaza, Stamford, CT 06902 USA; 2 Biostatisics Consult-Stat, Loyola Street, Macungie, PA 18062 USA; 3 Department of Medicine, Stamford Hospital, Columbia University College of Physicians and Surgeons, 1 Hospital Plaza, Stamford, CT 06902 USA; 4 Department of Critical Care Medicine, Faculty of Medicine and Dentistry, University of Alberta, 116 St. and 85 Ave, Edmonton, Alberta T6G 2R3 Canada

**Keywords:** Functional status, Critically ill, Mortality, Mortality prediction models, Acute physiology and chronic health evaluation IV

## Abstract

**Background:**

Functional status (FS) before intensive care unit (ICU) admission is associated with short-term and long-term outcomes among critically ill patients. However, measures of FS are generally not integrated into ICU-specific mortality prediction models.

**Methods:**

This retrospective cohort study used prospectively collected data from 9638 consecutive patients admitted to a single ICU between 1 October 2005 and 30 September 2015. For each ICU admission, FS was prospectively determined and classified into three discrete categories based on performance of basic daily living activities (FS1 - fully independent; FS2 - partly dependent; FS3 - completely dependent). We prospectively calculated Acute Physiology and Chronic Health Evaluation (APACHE) IV predicted mortality percentage (APIV PM) for each admission and calculated observed-expected mortality ratios (OEMR), stratified by FS category and APIV PM. We calculated area under the receiver operator characteristic curve (AUC) for APIV PM and mortality for the entire cohort and the three FS categories.

**Results:**

Patients had a median (IQR) age of 67 (52–80) years and mean (SD) APIV PM was 18.3% (24.3%). Of these, 7714 (80.0%) were classified as FS1, 1728 (17.9%) as FS2 and 196 (2.0%) as FS3. FS1 patients were younger, had less comorbid disease, and lower APIV PM compared to FS2 and FS3. The OEMR were significantly lower for FS1 (0.67) than FS2 (0.93) or FS3 (0.90) (*p* < 0.0001 for both comparisons). Among patients with APIV PM 0–10%, 10–25%, 25–50% and ≥50% the OEMR for FS1 were 0.33, 0.49, 0.61 and 0.86. The AUC (95% CI) for APIV PM and mortality for FS1, FS2 and FS3 were 0.924 (0.914–0.933), 0.837 (0.816–0.858) and 0.775 (0.705–0.8456), respectively (*p* < 0.001 for each comparison). Multivariable analysis demonstrated that FS2 (OR 2.18 (1.84–2.57) (p < 0.0001)) and FS3 (OR 1.99 (1.34–2.96) (p = 0.0006)) were independently associated with increased risk of mortality.

**Conclusions:**

Baseline FS prior to critical illness is a strong independent predictor of mortality and impacts the relationship between observed and APIV PM in those with lower illness severity. Future iterations of mortality prediction models should integrate a baseline measure of FS to improve performance.

**Electronic supplementary material:**

The online version of this article (doi:10.1186/s13054-017-1688-z) contains supplementary material, which is available to authorized users.

## Background

Mortality prediction models are a familiar component of critical care research and practice. They provide a validated metric to enable severity adjustment when mortality is evaluated as an endpoint in clinical investigations. Moreover, they are used for assessment of intensive care unit (ICU) performance over time and, less precisely, for case-mix adjusted benchmarking and administrative operational reporting [1].

Mortality prediction models, such as the Acute Physiology and Chronic Health Evaluation (APACHE) II, III and IV, Simplified Acute Physiology Score (SAPS), SAPS II, SAPS III, Mortality Prediction Model (MPM), MPM II and MPM III [1], include combinations of three critical domains: demographics, such as age and sex; chronic disease (medical comorbidities); and acute physiologic parameters - laboratory values and vital signs, typically obtained within the first 24 hours after ICU admission. Some also include admitting diagnosis (APACHE II, III, IV, SAPS III, MPM II and MPM III, operative or non-operative status (each of these except SAPS), origin of admission and time in the hospital preceding ICU admission (APACHE II, III, IV) [1]. Notably, preadmission functional status (FS) is not included in any of these models.

Preadmission functional status can be defined by capacity to perform the fundamental activities of daily living: transferring, bathing, dressing, feeding, personal hygiene and toileting [2]. Frailty has been recognized as an important contributor to FS and is a significant determinant of short-term and long-term prognosis for patients having an episode of critical illness [3–7].

While pre-hospital frailty prior to critical illness has been associated with increased hospital mortality, and among survivors, greater impairment in quality of life, incident disability and health services use [3–5], and while premorbid burden and trajectory in functional disability has been shown to predict worsening disability and death after critical illness [6], these measures have not been incorporated into commonly used mortality prediction models.

Accordingly, we developed and implemented a simple classification scheme to categorize pre-hospital FS for all patients admitted to our ICU, considering performance with the basic activities of daily living (fully independent, partially dependent and completely dependent), and place of residence (home, assisted living facility, skilled nursing facility or rehabilitation center). We hypothesized that stratifying patients based on preadmission FS would impact the performance of APACHE IV predicted hospital mortality (APIV PM).

## Methods

The Stamford Hospital Institutional Review Board approved this study. The need for informed consent was waived.

### Design, setting and population

This was a retrospective evaluation of prospectively collected data, abstracted from an ICU-specific administrative/operational database that included consecutive patient admissions from 1 October 2005 to 30 September 2015.

Stamford Hospital is a university-affiliated teaching hospital. The 16-bed ICU provides care for a wide case-mix of critically ill medical, surgical (including neurosurgical and cardiovascular) and major trauma patients. The hospital does not perform organ transplantation. The typical nurse-patient ratio in the ICU is 1:1 or 2:1, depending on the patient’s care requirements. Medical and surgical residents write orders in the ICU, closely supervised by a team of medical and surgical intensivists.

### Operational definitions

APIV PM is a comprehensive metric that includes a large number of physiologic parameters obtained during the first 24 hours of ICU admission, and age, origin of admission, admitting diagnosis to the ICU, mechanical ventilation and important medical comorbidities [8]. We have listed all of the components in this model in Additional file 1.

Functional status (FS) was assigned prospectively at the time of ICU admission by one investigator (JK), based on all available information from the medical record, patient and family members. The classification system includes three categories based on global assessment of performance of the basic activities of daily living - transferring, bathing, dressing, feeding, personal hygiene, and toileting (three designations: fully independent (FS1), partially dependent (FS2) and fully dependent (FS3)) and place of residence (three designations: home, assisted living facility and skilled nursing facility or rehabilitation facility). Mortality was defined as status at hospital discharge. One author (JK) prospectively calculated APIV PM (7).

### Data sources

The database includes detailed clinical information about each patient admission to the ICU since October 1998. One investigator (JK) has collected the core dataset, including demographics, comorbidities, admission and discharge time and date, admission diagnosis, severity of illness scores and metrics relating to mechanical ventilation. The database is linked to local hospital administrative data to capture detailed information from the laboratory, diagnostic imaging, costs and hospital discharge status.

### Statistical analysis

We report continuous data as median (interquartile range (IQR)) or mean (standard deviation (SD)) and compare groups using the Mann-Whitney rank sum test or Student’s *t* test, as appropriate. We report categorical data as numbers and percentages, and compare groups using the chi square test. We compared demographics, comorbidities, illness severity and clinical outcomes among the three FS groups.

We demonstrated the interaction of FS and the performance of the APIV PM model three ways. First, we calculated observed-expected mortality ratios (OE MR) as the quotient of observed hospital mortality and APIV PM and stratified results by severity of illness based on APIV PM using four groups, APIV PM <10%, APIV PM 10–25%, APIV PM 25–50% and APIV PM ≥50%, and by quintiles of APIV PM (Additional file 2). Second, we constructed receiver operator characteristic (ROC) curves and calculated the area under the ROC curve (AUC) for each FS, and further stratified this analysis by severity of illness based on APIV PM using two groups, APIV PM <10% and APIV PM ≥10%. We compared the AUC: (1) between groups of functional status levels for the entire cohort; (2) between patients with APIV PM <10% and ≥10%; and (3) for each FS, comparing those with APIV PM <10% to those with APIV PM ≥10%. We created calibration plots to further illustrate the relationship between observed and predicted mortality for the entire cohort and for the three FS categories [9]. Finally, we performed multivariable analysis including APIV PM to assess the independent association of FS with mortality, and a sensitivity analysis that evaluated this association among medical, surgical and trauma patients.

The Strengthening of Reporting in Observational studies in Epidemiology (STROBE) checklist was used to guide study design (Additional file 3) (http://www.strobe-statement.org/?id=available-checklists). Analyses were performed using the MedCalc program for statistical analysis (MedCalc Statistical Software version 15.4 (MedCalc Software bvba, Ostend, Belgium; https://www.medcalc.org; 2015).

## Results

A total of 10,149 patients were admitted to the ICU during the study period; 511 (5.0%) were excluded due to admission after cardiovascular surgery, as ICU admission for these patients did not include calculation of APIV PM. Table 1 details the clinical characteristics and outcomes of the three FS categories. Patients classified as FS1 were younger, had fewer comorbidities, were more likely to have a postoperative or trauma diagnosis and had less severe illness, shorter ICU stay and lower mortality compared to those classified as FS2 or FS3. The OE MR was lower for FS1 compared with both FS2 and FS3 (*p* < 0.001 for both comparisons).

**Table 1:** Clinical characteristics of the patients

	FS1	FS2	FS3	FS1 vs FS2	FS1 vs FS3
Number	7714	1728	196		
Domicile					
Home	7644	932	87		
Assisted living	31	116	9		
Rehabilitation/SNF	38	688	100		
Age	64 (49–77)	80 (70–86)	75 (56–86)	<0.0001	<0.0001
Charlson	1 (0–2)	2 (1–4)	2 (1–3)	<0.0001	<0.0001
Diagnostic category (%)					
Medical	56.2	78.4	89.8	<0.0001	<0.0001
Surgical	31.2	16.0	9.2	<0.0001	<0.0001
Trauma	12.6	5.6	1.0	<0.0001	<0.0001
Diabetes mellitus (%)	19.7	29.3	22.2	<0.0001	0.4371
APACHE II comorbidities (%)					
Pulmonary	5.4	17.1	31.6	<0.0001	<0.0001
Cardiac	7.1	22.2	13.3	<0.0001	0.0015
End-stage renal disease	2.6	8.7	2.0	<0.0001	0.7681
Portal hypertension	2.3	2.0	1.5	0.6973	0.6180
Metastatic cancer	6.5	9.3	3.6	0.0051	0.1369
APACHE II score	12 (8–18)	20 (15–26)	22 (18–28)	<0.0001	<0.0001
APACHE IV score	44 (31–63)	69 (54–90)	75 (56–94)	<0.0001	<0.0001
APIV PM (%)	14.8 (22.2)	32.0 (27.4)	32.8 (24.7)	<0.0001	<0.0001
Ventilation (%)	30.5	43.8	59.2	<0.0001	<0.0001
ICU LOS	1.4 (0.8–2.8)	1.9 (1.0–4.4)	2.5 (1.2–6.6)	<0.0001	<0.0001
Mortality (%)	9.9	29.7	29.6	<0.0001	<0.0001
OE MR	0.67	0.93	0.90	<0.0001	<0.0001

*FS* functional status*, FS1* independent in performing activities of daily living (ADL), *FS2* partly dependent in performing ADL, *FS3* fully dependent in performing ADL, *SNF* skilled nursing facility, *APIV PM* Acute Physiology and Chronic Health Evaluation IV (APACHE IV) predicted mortality, *LOS* length of stay, *OE MR* observed-expected mortality ratio (using hospital discharge status and APIV PM)

Table 2 reports OE MR for patients stratified by FS and APIV PM. The difference between OE MR for FS1 compared to FS2 and FS3 was greatest for patients in the lowest strata of APIV PM. As APIV PM increased, this difference decreased; in patients with the most severe illness, OE MR were similar for the three FS groups.

**Table 2:** Observed-expected mortality ratios, stratified by FS and APIV PM

Cohort	Number	Mortality (%)	APIV PM (%)	OE MR
APIV PM <10%				
FS1	5054	1.1	3.3	0.33
FS2	434	5.1	5.6	0.91
FS3	41	4.9	4.9	1.00
APIV PM <10–25%				
FS1	1279	7.9	16.1	0.49
FS2	490	16.1	16.8	0.96
FS3	49	20.4	17.0	1.20
APIV PM <25–50%				
FS1	671	21.3	35.1	0.61
FS2	411	32.1	36.3	0.89
FS3	62	29.0	37.2	0.78
APIV PM ≥50%				
FS1	710	65.2	75.4	0.86
FS2	393	71.5	75.8	0.94
FS3	44	63.6	70.1	0.91

*APIV PM* Acute Physiology and Chronic Health Evaluation IV (APACHE IV) predicted mortality (%), *OE MR* observed-expected mortality ratio. Statistical testing for differences between FS for OE MR:APIV PM <10%; FS1 vs FS2 *p* < 0.0001; FS1 vs FS3 *p* < 0.0001. APIV PM <10–25%; FS1 vs FS2 *p* < 0.0001; FS1 vs FS3 *p* < 0.0001. APIV PM <25–50%; FS1 vs FS2 *p* < 0.0001; FS1 vs FS3 *p* = 0.0082. APIV PM ≥50%; FS1 vs FS2 *p* = 0.0001; FS1 vs FS3 *p* = 0.3494

**Fig. 1:**
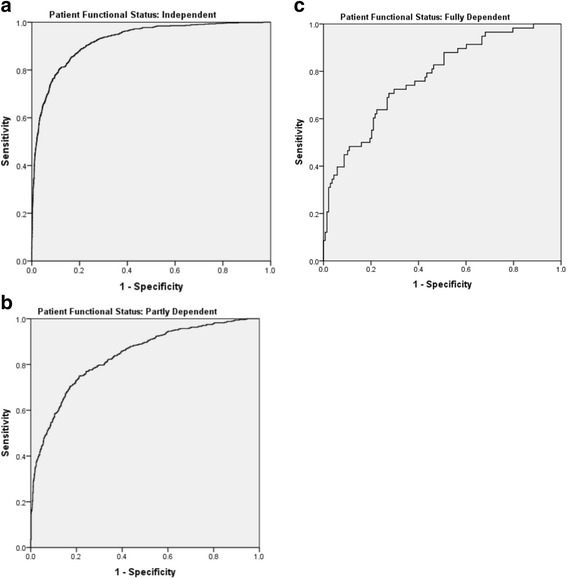
**a** Receiver operator characteristic (*ROC*) curve for functional status 1 (*FS1*). **b** ROC curve for FS2. **c** ROC curve for FS3

**Fig. 2:**
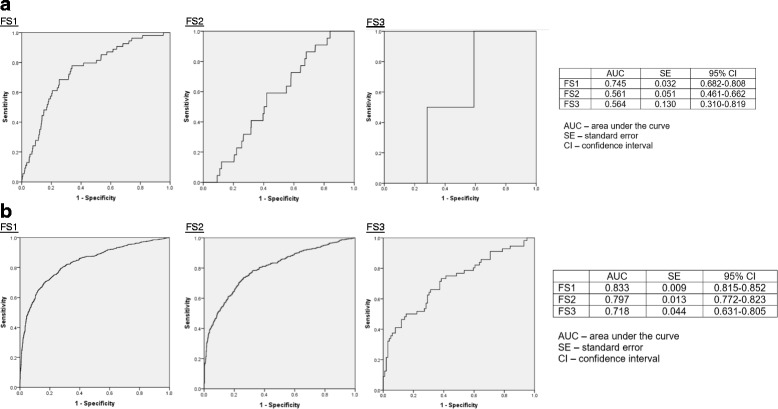
**a** Receiver operator characteristic (*ROC*) curves for patients with Acute Physiology and Chronic Health Evaluation IV predicted mortality (*APIV PM*) predicted mortality <10%. **b** ROC curves for patients with APIV PM ≥10%. *AUC* area under the curve, *FS* functional status

Figure 1 displays the ROC curves for APIV PM stratified by FS group. Tables 3, 4, 5 and 6 detail the AUC of these curves, demonstrating significantly greater AUC for FS1 compared to the other groups, and Fig. 2 displays greater AUC for FS1 and FS2 among patients with APIV PM ≥10% compared to those with APIV PM <10%.

**Table 3:** Comparisons of area under the curve between patients grouped by functional status

					*P* values for comparison between functional status groups		
Functional status	Dead	Alive	Area	SE	Independent	Partly dependent	Fully dependent
Independent	761	6953	0.924	0.005	1.0		
Partly dependent	514	1214	0.837	0.011	<0.001	1.0	
Fully dependent	58	138	0.775	0.036	<0.001	0.074	1.0

**Table 4:** Comparisons of area under the curve between patients grouped by functional status - patients with APACHE IV predicted mortality <10%

					*P* values for comparison between functional status groups		
Functional status	Dead	Alive	Area	SE	Independent	Partly dependent	Fully dependent
Independent	54	5001	0.745	0.032	1.0		
Partly dependent	22	412	0.561	0.051	0.005	1.0	
Fully dependent	2	39	0.564	0.130	0.374	0.989	1.0

*APACHE* Acute Physiology and Chronic Health Evaluation

**Table 5:** Comparisons of area under the curve between patients grouped by functional status - patients with APACHE IV predicted mortality ≥10%

					*P* values for comparison between functional status groups		
Functional status	Dead	Alive	Area	SE	Independent	Partly dependent	Fully dependent
Independent	707	1952	0.833	0.009	1.0		
Partly dependent	492	802	0.797	0.013	0.012	1.0	
Fully dependent	56	99	0.718	0.044	0.006	0.063	1.0

*APACHE* Acute Physiology and Chronic Health Evaluation

**Table 6:** Comparisons of area under the curve between patients grouped by functional status and predicted mortality <10% and ≥10%

Functional status	<10%		≥10%		*P* value
	Area	SE	Area	SE	
Independent	0.745	0.032	0.833	0.009	0.002
Partly dependent	0.561	0.051	0.797	0.013	<0.001
Fully dependent	0.564	0.130	0.718	0.044	0.464

Additional file 4: Figure S1a-h displays calibration plots for the entire cohort and for each FS group. These results indicate that for the entire sample including all functional status levels there is a high degree of calibration within these data.

Table 7 reports the results of multivariable analysis of FS and APIV PM for mortality, demonstrating that FS2 and FS3 are independently associated with increased risk of death among the entire cohort and in the medical and surgical subpopulations. The point estimate of the OR for mortality for medical, surgical and trauma patients is nearly identical.

**Table 7:** Multivariable analysis, including sensitivity analysis: independent association of FS with mortality

Cohort	OR (95% CI)	*P* value
Entire cohort		
FS2 vs FS1	2.18 (1.84–2.57)	<0.0001
FS3 vs FS1	1.99 (0.34–2.96)	0.0006
Medical patients		
FS2 vs FS1	2.20 (1.83–2.65)	0.0001
FS3 vs FS1	2.07 (1.38–3.10)	0.0004
Surgical patients		
FS2 vs FS1	2.04 (1.28–3.26)	0.0029
FS3 vs FS1	1.60 (0.26–9.85)	0.6149
Trauma patients		
FS2 vs FS1	2.20 (0.96–5.08)	0.0640
FS3 vs FS1^a^	N/A	

Multivariable analysis includes Acute Physiology and Chronic Health Evaluation IV predicted mortaltiy. *FS* functional status. ^a^Only two trauma patients had FS3

## Discussion

Our large retrospective study capturing prospective pre-hospital functional status data on consecutive admissions to a large academic ICU provides important insights into the impact of baseline function on outcome after critical illness and the performance of standard illness severity scoring systems to predict mortality.

### Key findings

The salient finding of this investigation is that preadmission FS is independently associated with the probability of mortality among critically ill patients, impacting the performance of the APACHE IV model of prediction of mortality. We demonstrate the interaction of FS and the APACHE IV model: (1) by analyzing observed-expected mortality ratios, stratified by FS and severity of illness; (2) by creating ROC curves for the three FS; and (3) by reporting the results of multivariable analysis. This interaction was robust across all three analyses. The independent association of preadmission FS with mortality is most evident in patients admitted to the ICU with low severity of illness, and diminishes with increasing severity of illness. Patients with impaired preadmission FS sustained higher mortality than predicted for lower severity scores, suggesting that the APACHE IV model may underestimate risk in these patients. Among patients admitted with high acuity illness, reflected by APACHE IV predicted mortality >50%, the independent impact of preadmission FS is vitiated. This finding is not modified by diagnostic category (medical, surgical or trauma service admission).

We note that impaired baseline FS was relatively uncommon in this cohort of ICU patients and largely driven by those classified as partially dependent (close to one in five) due to impairment in ADL, more than half of whom were still living at home. ICU admission for those with severely impaired status (skilled nursing facility (SNF) and full dependence) was exceedingly uncommon, representing approximately 1% of the entire cohort, suggesting a strong element of preadmission triage and selection. The subgroup classified as FS3 were predominantly medical patients, with greater prevalence of co-existing disease, highest utilization of mechanical ventilation, and longer duration of ICU stay. In contrast, the shortest ICU stay and lowest mortality and O-E mortality was observed for those with preserved preadmission FS.

### Context with prior literature

Emerging literature has described the association of pre-hospital FS with short-term outcomes - specifically, hospital mortality - and longer-term functional outcomes [3–6]. This is biologically plausible. Ferrante and coworkers performed a prospective investigation of 754 persons 70 years or older, 291 of whom required ICU admission, to evaluate the relationship between preadmission FS and short-term and long-term outcome after ICU illness. Persons with mild to moderate disability before admission had more than double the risk of death within one year of ICU admission, and increased ICU LOS, mechanical ventilation and shock. For persons with severe preadmission disability the risk of death within one year of ICU admission was nearly fourfold higher [6]. Similarly, a multicenter French investigation of 196 patients ≥65 years old evaluated the relationship between frailty, determined at the time of ICU admission and ICU outcome [5]. Notably, while there was no difference in severity of illness scores (SAPS II, Sequential Organ Failure Assessment (SOFA)) comparing patients with and without frailty, the presence of frailty was independently associated with increased risk of morbidity and mortality. Moreover, Baldwin and coworkers evaluated medical records and claims data for 1565 patients aged ≥65 years who were admitted to a single tertiary center ICU in order to create a 6-month post-discharge mortality model [7]. Admission from an SNF, a surrogate for frailty and preadmission FS, was independently associated with 6-month post-discharge mortality (OR 2.39 (95% CI 1.73–3.30), *p* < 0.001). In addition, the Charlson comorbidity score was also strongly associated with post-discharge mortality. Compared to patients with model prediction scores 0–1, the OR (95% CI) for those with scores 2–5, 6–7 and ≥8 were 1.85 (1.38–2.47, *p* < 0.001), 2.30 (1.32–4.00, *p* = 0.003) and 7.20 (3.33–15.50, *p* < 0.001), respectively. These data were largely corroborated by Parlevliet et al., who found that health-related quality of life (HRQOL, utility based on the EuroQol-5D score) at the time of ICU admission was independently associated with risk of mortality and functional decline in a cohort of patients ≥65 years old admitted to three hospitals in the Netherlands [10], and Zeng et al. who evaluated the relationship between a frailty index based on 52 discrete acute and chronic characteristics, and 300-day post-discharge mortality in a cohort of older patients admitted to a single geriatric ICU in China [11]. Finally, Bagshaw and coworkers used a validated “global” measure of frailty to demonstrate the independent effect of impaired preadmission FS on hospital and on one-year post-discharge mortality and functional outcomes [12]. Frailty, as assessed by the Clinical Frailty Scale, was independently associated with hospital mortality (OR 1.81 (95% CI 1.09–3.01)) and one-year mortality (OR 1.82 (95% CI 1.28–2.60)), and with greater risk of developing functional dependence after hospital discharge and being readmitted to the hospital.

To our knowledge, there are no previous investigations that have described the impact of preadmission FS on the performance of mortality prediction models. Mortality prediction models include an array of clinical parameters present at the time of ICU admission that are used to derive prediction of hospital mortality [1]. The most widely used model, the APACHE II, includes 12 physiologic variables, and age, surgical status, admission diagnosis and a small group of important medical comorbidities [12]. Other models, such as the MPM [13], APACHE III [14], SAPS II [15], MPM II [16], SAPS 3 [17], MPM III [18] and APACHE IV [8], found to have the highest precision among the models [19, 20], include different numbers of physiologic parameters, age, surgical status and various arrays of medical comorbidities. Specifically, the medical comorbidities that contribute to increased risk of mortality in the APACHE II and IV models include, respectively: Class IV cardiac or pulmonary disease, portal hypertension, end-stage renal disease, metastatic cancer and immunosuppression for APACHE II and cirrhosis, lymphoma, leukemia and multiple myeloma, immunosuppression, hepatic failure, metastatic cancer and the acquired immune deficiency syndrome for APACHE IV. These comorbidities are a surrogate for chronic health status but their presence does not necessarily correlate with a patient’s functional capacity. Notably, none of the mortality prediction models includes a metric that describes preadmission FS.

### Implications for practice, policy, and future research

We contend that our findings demonstrating that preadmission FS confounds the precision of the APACHE IV mortality prediction model, in particular among patients with low severity of illness, have implications for intensive care professionals and policy makers. First, ICU providers should recognize the incremental risk of less favorable outcomes and greater resource utilization for patients with impaired FS. This may prompt ICU clinicians to consider, earlier in the course of ICU illness, discussions with families regarding goals of care, including but not limited to patient and family preferences relating to the intensity and duration of ICU support, in particular among those with a pre-hospital decline trajectory [6]. Second, importantly, we have shown that among those with lower illness severity and impaired baseline FS, the illness severity model predicted mortality may grossly underestimate true risk. This impact of preadmission FS should inform the interpretation of unit-level survival statistics and external benchmarking.

Future research should aim to replicate our findings utilizing a similar measure of FS or alternative validated measure across currently used mortality prediction tools for ICU patients. Data repositories for ICU patients should include components describing preadmission FS; ideally, these metrics should be standardized to allow benchmarking and facilitate research efforts. One notable example of such an effort is the Intensive Care National Audit and Research Center (ICNARC) database, aggregating outcomes of ICU patients from over 200 institutions in the UK [21]. Similarly, future iterations of mortality prediction models should aim to integrate a readily accessible and reliable measure of pre-hospital FS.

### Limitations

Our study has several notable limitations. First, our study was retrospective and single-center; however, we prospectively captured baseline FS and illness severity scores for all ICU admissions during the study period. Second, our study did not capture detailed information on patients referred to ICU and refused admission, potentially predisposing to an element of selection bias. Third, we used a relatively crude assessment of pre-hospital FS compared to prior investigations [3]. However, we contend that this FS classification scheme can be easily determined at the time of ICU admission.

## Conclusions

Preadmission functional status is independently associated with mortality among critically ill patients. Future iterations of mortality prediction models should include in their design a metric that describes preadmission functional status.

## Additional files

Additional file 1: Components of the APACHE IV mortality prediction model (DOC 24 kb)

Additional file 2: Observed-expected mortality ratios, stratified by FS and severity of illness. Observed-expected mortality ratios for each FS, stratified by quintile of APACHE IV predicted mortality (DOC 54 kb)

Additional file 3: STROBE statement - checklist of items that should be included in reports of cohort studies (DOC 82 kb)

Additional file 4: Calibration plots for the entire cohort and for each FS, with and without scatter. The document provides eight separate figures: Calibration plots for the entire cohort and for each FS, with and without scatter, and additional text describing calibration plots and the enclosed data (DOCX 171 kb)

## Abbreviations

ADL: Activities of daily living
APACHE: Acute Physiology and Chronic Health Evaluation
APIV PM: Acute Physiology and Chronic Health Evaluation IV predicted mortality
AUC: area under the curve
FS: Functional status
HRQOL: Health related quality of life
ICU: Intensive care unit
LOS: Length of stay
MPM: Mortality Prediction Model
OE MR: Observed-expected mortality ratio
ROC: receiver operating characteristic
SAP: Simplified Acute Physiology Score
SNF: Skilled nursing facility
SOFA: Sequential Organ Failure Assessment
