# Supplementary material for: Pre-admission functional status impacts the performance of the APACHE IV model of mortality prediction in critically ill patients
Source: Crit Care. 2017 May 15;21:110. doi: 10.1186/s13054-017-1688-z (PMC5433010; doi:10.1186/s13054-017-1688-z)
Supplement: Supplementary file 1 — Components of the APACHE IV mortality prediction model (DOC 24 kb) [file 13054_2017_1688_MOESM1_ESM.doc]

Additional file 1

Components of the APACHE IV mortality prediction model

Physiologic parameters:

- Rectal temperature
- Mean arterial pressure
- Heart rate
- Respiratory rate
- A-aDO2 (FiO2 > 0.5) or PaO2 (FiO2 < 0.5)
- Serum Na
- Glucose
- Hematocrit
- WBC coung
- Serum Cr (2 scales – with and without acute kidney injury)
- Urine output
- BUN
- Bilirubin
- Albumin
- Acid base status, using a grid that includes pH and PaCO2

Neurologic performance, based on the components of the Glasgow Coma Scale

Age

Chronic health conditions:

- AIDS
- Hepatic failure
- Lymphoma
- Metastatic cancer
- Leukemia/multiple myeloma
- Immunosuppression
- Cirrhosis
