# Supplementary material for: Pre-admission functional status impacts the performance of the APACHE IV model of mortality prediction in critically ill patients
Source: Crit Care. 2017 May 15;21:110. doi: 10.1186/s13054-017-1688-z (PMC5433010; doi:10.1186/s13054-017-1688-z)
Supplement: Supplementary file 2 — Observed-expected mortality ratios, stratified by FS and severity of illness. Observed-expected mortality ratios for each FS, stratified by quintile of APACHE IV predicted mortality (DOC 54 kb) [file 13054_2017_1688_MOESM2_ESM.doc]

Additional file 2 Observed:expected mortality ratios, stratified by FS and severity of illness

| Cohort | Number | Mortality (%) | APIV PM (%) | OEMR |
| --- | --- | --- | --- | --- |
| Quintile 1 |  |  |  |  |
| FS1 | 1543 | 0.26 | 0.87 | 0.30 |
| FS2 | 346 | 5.49 | 4.71 | 1.17 |
| FS3 | 40 | 5.00 | 4.75 | 1.05 |
|  |  |  |  |  |
| Quintile 2 |  |  |  |  |
| FS1 | 1543 | 0.52 | 2.30 | 0.23 |
| FS2 | 346 | 10.98 | 12.28 | 0.89 |
| FS3 | 39 | 20.51 | 15.01 | 1.37 |
|  |  |  |  |  |
| Quintile 3 |  |  |  |  |
| FS1 | 1543 | 1.88 | 5.18 | 0.36 |
| FS2 | 346 | 17.92 | 22.96 | 0.78 |
| FS3 | 39 | 20.51 | 27.93 | 0.73 |
|  |  |  |  |  |
| Quintile 4 |  |  |  |  |
| FS1 | 1543 | 6.61 | 13.32 | 0.50 |
| FS2 | 345 | 39.71 | 41.53 | 0.96 |
| FS3 | 39 | 35.90 | 44.44 | 0.81 |
| Quintile 5 |  |  |  |  |
| FS1 | 1542 | 40.08 | 52.47 | 0.76 |
| FS2 | 345 | 74.78 | 78.86 | 0.95 |
| FS3 | 39 | 66.67 | 72.49 | 0.92 |
