# Supplementary material for: Pre-admission functional status impacts the performance of the APACHE IV model of mortality prediction in critically ill patients
Source: Crit Care. 2017 May 15;21:110. doi: 10.1186/s13054-017-1688-z (PMC5433010; doi:10.1186/s13054-017-1688-z)

Additional file 4

Calibration Plots for the entire cohort as well as for each FS, with and without scatter

Calibration plots were calculated using the methodology of Vuk and Curk (2006). Calibration is calculated by comparing the predicted value for individual patients against the observed outcome variable, in this case mortality. The diagonal line included in the plot represents the situation where prediction perfectly matched observed outcome. As can be seen in the calibration plot for all functional status levels combined there is a near perfect fit across all areas of the probabilities spectrum 0.0 to 1.0. Similarly if bias were present in the calibration plot the LOESS regression spline would be above or below the diagonal line across all observed variable scale. As can be seen there is no bias present in the calibration provided. While predicted values tend to be lower than observed data in the lower 40% of the distribution the regression spline crosses the diagonal and nearly returns to perfect prediction in the upper portion of the scale observed variable. These results indicate that for the entire sample including all functional status levels there is a high degree of calibration within these data.

**Reference:**

Vuk M, Curk T. ROC Curve, Lift Chart and Calibration Plot, Metodoloski zvezki 2006:3:1:89-108

Figure S1a: Calibration Plot for the entire cohort, without scatter


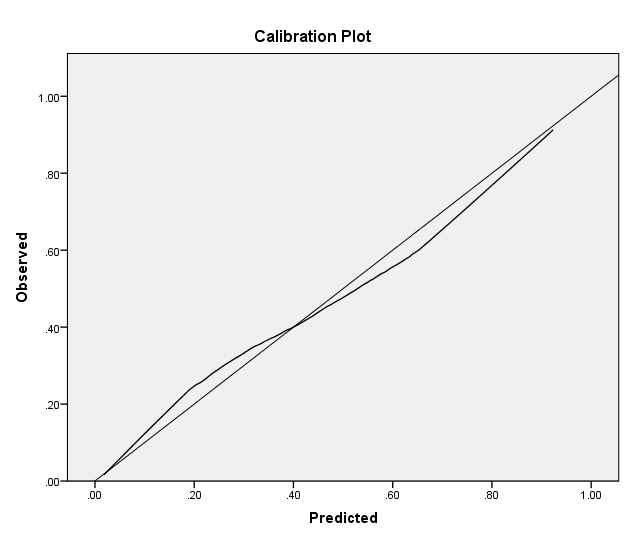


Figure S1b: Calibration Plot for the entire cohort, with scatter


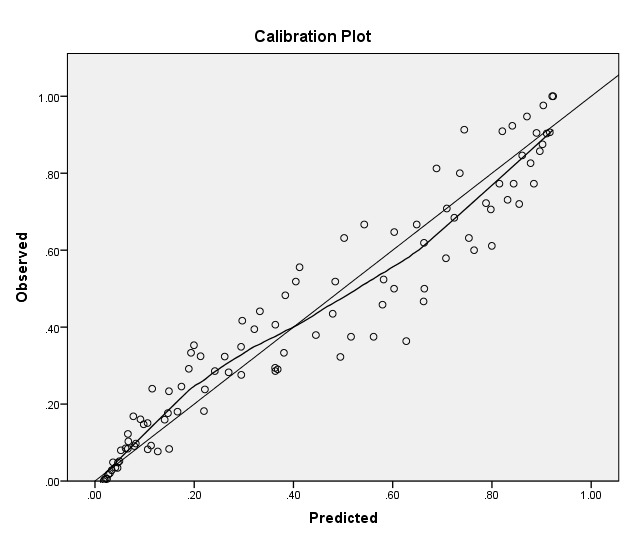


Figure S1c: Calibration Plot without scatter for FS1


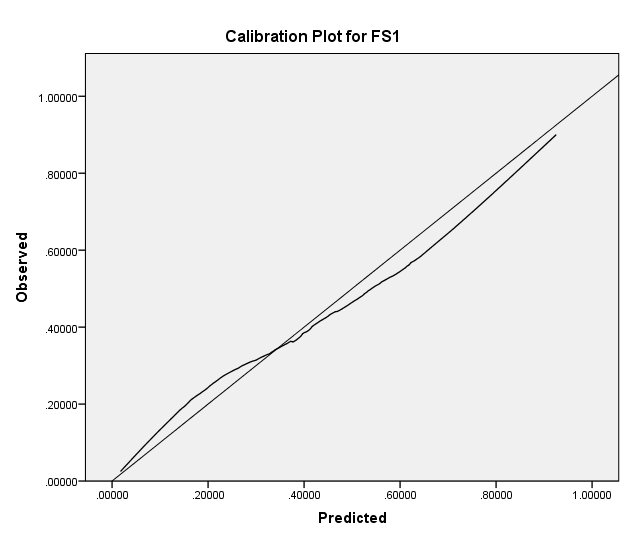


Figure S1d: Calibration Plot with scatter for FS1


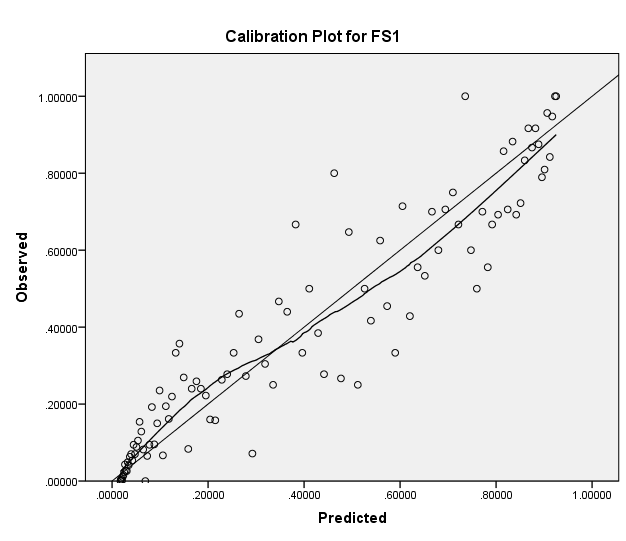


Figure S1e: Calibration Plot without scater for FS2


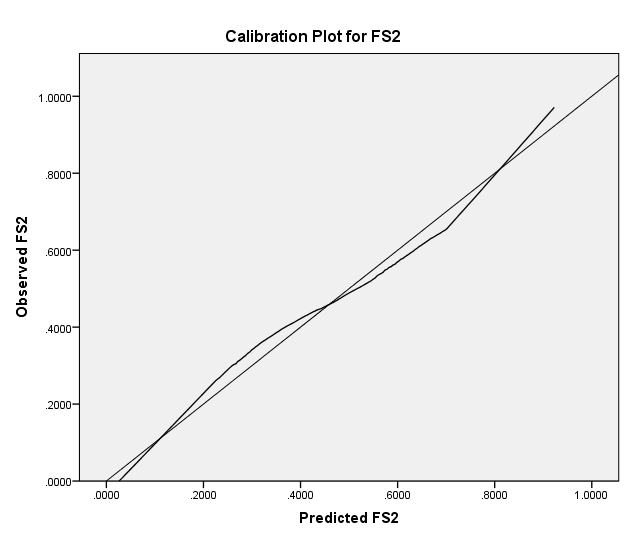


Figure S1f: Calibration Plot with scatter for FS2


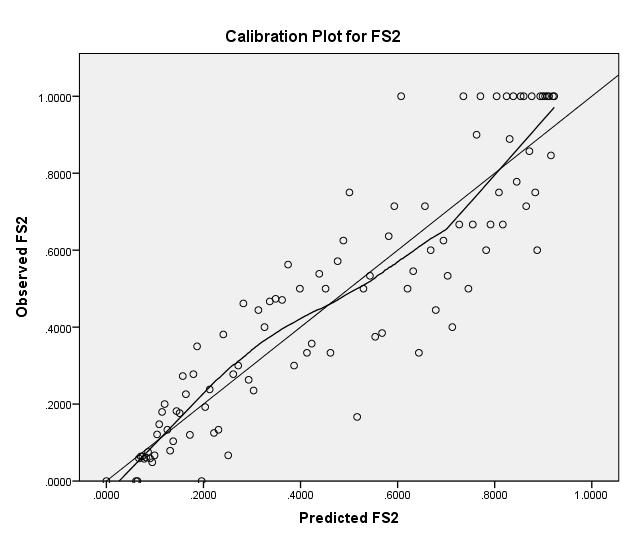


Figure S1g: Calibration Plot without scatter for FS3


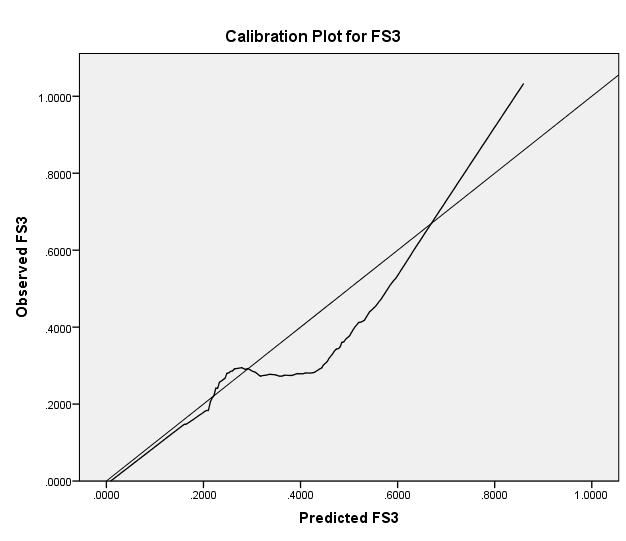


Figure S1h: Calibration Plot with scatter for FS3
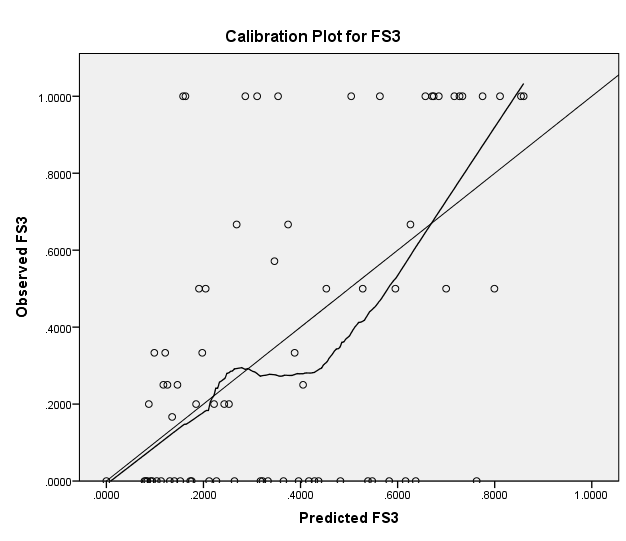

Supplement: Supplementary file 4 — Calibration plots for the entire cohort and for each FS, with and without scatter. The document provides eight separate figures: Calibration plots for the entire cohort and for each FS, with and without scatter, and additional text describing calibration plots and the enclosed data (DOCX 171 kb) [file 13054_2017_1688_MOESM4_ESM.docx]
